# Construction of the influenza A virus transmission tree in a college-based population: co-transmission and interactions between influenza A viruses

**DOI:** 10.1186/s12879-016-1373-x

**Published:** 2016-01-29

**Authors:** Xu-Sheng Zhang, Daniela De Angelis

**Affiliations:** 1 Centre for Infectious Disease Surveillance and Control, Public Health England, 61 Colindale Avenue, London, NW9 5EQ UK; 2 Medical Research Council Centre for Outbreak Analysis and Modelling, Department of Infectious Disease Epidemiology, Imperial College School of Public Health, Norfolk Place, London, W2 1PG UK; 3 Medical Research Council Biostatistics Unit, University Forvie Site, Robinson Way, Cambridge, CB2 0SR UK

**Keywords:** Co-infection, Co-transmission, Inference, Influenza, Outbreak, Strain interactions

## Abstract

**Background:**

Co-infection of different influenza A viruses is known to occur but how viruses interact within co-infection remains unknown. An outbreak in a college campus during the 2009 pandemic involved two subtypes of influenza A: persons infected with pandemic A/H1N1; persons infected with seasonal A/H3N2 viruses; and persons infected with both at the same time (co-infection). This provides data to analyse the possible interaction between influenza A viruses within co-infection.

**Methods:**

We extend a statistical inference method designed for outbreaks caused by one virus to that caused by two viruses. The method uses knowledge of which subtype each case is infected with (and whether they were co-infected), contact information and symptom onset date of each case in the influenza outbreak. We then apply it to construct the most likely transmission tree during the outbreak in the college campus.

**Results:**

Analysis of the constructed transmission tree shows that the simultaneous presence of the two influenza viruses increases the infectivity and the transmissibility of A/H1N1 virus but whether it changes the infectivity of A/H3N2 is unclear. The estimation also shows that co-transmission of both subtypes from co-infection is low and therefore co-infection cannot be sustained on its own.

**Conclusions:**

This study suggests that influenza A viruses within co-infected patients can interact in some ways rather than transmit independently, and this can enhance the spread of influenza A virus infection.

**Electronic supplementary material:**

The online version of this article (doi:10.1186/s12879-016-1373-x) contains supplementary material, which is available to authorized users.

## Background

Co-circulation of multiple types and subtypes of influenza virus has been commonly observed in human populations [1, 2]. With advanced molecular techniques [3, 4], it is now becoming possible to rapidly detect different subtypes or strains of a disease agent within infected patients. There is accumulating evidence to show that the phenomenon that multiple subtypes of influenza A virus infect an individual simultaneously (i.e. co-infection) is not as rare as we previously thought [5–10]. Hence it is interesting to know how the simultaneous presence of two strains alters, compared to singly infected individuals, the transmissibility of each subtype and of both subtypes together. Some observations of transmission involved with co-infected individuals have been reported [11, 12]. One important observation reported is individuals co-infected with pandemic A/H1N1 and seasonal A/H3N2 influenza viruses within one outbreak at a college campus in Beijing, China during the 2009 pandemic [12]. Liu et al. [12] provides direct evidence that individuals co-infected with different subtypes can transmit viruses separately or simultaneously and provides detailed data for us to quantify the interactions between virus strains. The transmission characteristics can be directly estimated from the transmission tree; however, the difficulty for constructing the transmission tree is that some contacts were missed. Fortunately, recent development in statistical inference [13, 14] allows us to construct the transmission tree of a single influenza A virus from such a partial contact network. In this short report we extend this inference method to construct the transmission tree that includes two influenza A viruses and their co-infection. From the constructed tree we estimate the impact of co-infection on transmission. We use two parameters to characterise the impact: the co-transmission rate and an interaction parameter. Here we define the former as the rate at which two strains simultaneously transmit from doubly infected individuals to susceptible individuals; and the latter as the ratio of transmissibility of each single virus from co-infected individuals to the transmissibility of each single virus from singly infected individuals.

### Data

Here we briefly summarize the data collection method and data of Liu et al. [12]. Investigations were conducted on all influenza like illness (ILI) cases identified during the outbreak. Epidemiological, clinical and contact tracing data were collected by interviewing patients and retrieving medical records. Viruses were identified by reverse-transcription polymerase chain reaction assays followed by sequence analysis. The heamagglutination inhibition tests were used to detect antibodies to both viruses. The outbreak is reported to have taken place within three buildings (two dormitories and one college clinic). No other cases at the college were reported. Buildings 1 and 2 (with a total membership of 235 and 191 persons, respectively) are next to each other and there is restricted access between the two and to the wider community. Forty five ILI cases were reported from 31 August to 10 September and forty (*N* = 40) had laboratory-confirmed influenza A infection. Three different types of infection were reported: 22 infected with pandemic A/H1N1 virus, 12 infected with seasonal A/H3N2 virus and six co-infected with both influenza A viruses. In their sequences no substantial differences were observed between patients with mixed and single infections in either pandemic A/H1N1 or seasonal A/H3N2 virus. The clinical features were similar for patients with different infections and the six co-infected patients showed no more severe symptoms than the singly infected patients. Contacts between infected people are shown in Fig. 2 of Liu et al. [12] but this only extends to the contact network within one dormitory. The ‘index’ case with pandemic influenza A/H1N1 infection was a college student whose symptoms first occurred on 31 August, 2 days after his returning to college. Except for the index case, all patients with A/H1N1 infection had not left the campus during the previous week. In contrast, the source of seasonal A/H3N2 virus infection cannot be determined exactly although available data indicate that A/H3N2 virus might have been prevailing in the college when the pandemic H1N1 virus was introduced. Before the isolation of cases and the initiation of prophylaxis among the campus population (5 September 2009), several patients visited the college clinic and the mixing between students of different dormitories was not frequent in comparison to the mixing between students within each dormitory.

## Methods

For a fully traced transmission tree (i.e., the information of the infector **v** and time of symptom onset *t* are collected), the infector *v*(*i*) for each case *i* (except the index case) and the duration between symptom onset of case *i* and symptom onset of its infector *v*(*i*): *t*_*i*_-*t*_*v*(*i*)_ should be known. From these it is straightforward to estimate the generation interval distribution and transmissibility of infection. In reality, however, it is rare and difficult to record all the information. Based on the partially known contact tracing data and dates of symptom onset, Hens et al. [14] illustrated an inference method to reconstruct the most likely transmission tree that involved with only one virus. Here we further develop it to a transmission tree during an outbreak that involves with two viruses of similar epidemiological characteristics.

In general, a possible transmission tree can be described by *p*_*ij*_(**v**,**w,φ;θ**), the probability that case *j* is the infector of case *i*, given the duration between symptom onset of case *i* and case *j*, given the information on the possible infector **v** and the known contacts **w**, and given the types **φ** of infection of both cases. Following Hens et al [14], its total log-likelihood is given by:

1 $$ L\left(\boldsymbol{\uptheta} \mathbf{\Big|}t,\mathbf{v},\mathbf{w},\boldsymbol{\upvarphi} \right)={\displaystyle \sum_{i=2}^N{\displaystyle \sum_{j=1}^N{p}_{ij}\left(\mathbf{v},\mathbf{w},\boldsymbol{\upvarphi}; \boldsymbol{\uptheta} \right) \log \left[g\left({t}_i-{t}_j\Big|\boldsymbol{\uptheta} \right)\right]}} $$

The sum runs through all the non-zero *p*_*ij*_. Here g(∆*t* |**θ**) denotes the probability density of the generation interval distribution of influenza infection, with **θ** representing the set of parameters that characterise the probability density distribution. Different distributions such as gamma, log-normal and Weibull can be used to describe the distribution of generation intervals [14]. Here we assume it follows a Weibull distribution:

2 $$ \begin{array}{l}g\left(\varDelta t\Big|\boldsymbol{\uptheta} \right)=\frac{b}{a}{\left(\frac{\varDelta t}{a}\right)}^{b-1} \exp \left(-{\left(\frac{\varDelta t}{a}\right)}^b\right)\kern0.5em \mathrm{when}\kern0.5em \varDelta t\ge 0,\\ {}g\left(\varDelta t\Big|\boldsymbol{\uptheta} \right)=0\kern0.5em \mathrm{otherwise}.\end{array} $$

The distribution has two parameters (i.e., **θ** = {*a*, *b*}): scale parameter *a* and shape parameter *b*, such that the mean is *T* = *a*Γ(1 *+* 1*/b*) and the variance is *σ* ^2^ = *a* ^2^[Γ(1 *+* 2*/b*)- Γ(1 *+* 1*/b*)^2^]. Here Γ() is the Gamma function.

Compared with outbreaks that involved one virus and one transmission process [14], this outbreak [12] involved two viruses and five possible transmission processes: from A/H1N1 to A/H1N1; from A/H3N2 to A/H3N2; from co-infection to A/H1N1; from co-infection to A/H3N2 and from co-infection to co-infection. However, the data given in Liu et al. [12] only provide the relevant information for three transmission processes: from A/H1N1 to A/H1N1; from co-infection to A/H1N1; and from co-infection to co-infection. The simple calculations show that the mean generation intervals (and their standard deviations) for the three transmission processes are 1.8 (0.8), 1.2 (0.4), 1.6 (0.6) days, respectively. In this data there is no evidence that they differ. As no data is available for the transmission processes that were involved with A/H3N2 [12], it is difficult to estimate their generation intervals and judge how they differ from those generation intervals involved with pandemic A/H1N1. Nevertheless, a recent systematic review [15] indicates that the generation interval of pandemic A/H1N1 virus was similar to that of the seasonal flu. Further, as observed by Liu et al. [12], the three different types of infections have similar epidemiological characteristics. In view of these, the same generation interval distribution is assumed for the three types of infection.

The probability that case *i* is infected by case *j*, *p*_*ij*_, can be calculated as the probability of observing the duration between the symptom onsets in cases *i* and *j*, g(*t*_i_-*t*_j_|**θ**), times the probability of a potentially infectious contact between *i* and *j*, *π*_*ij*_, normalized by the probability of *i* being infected by any other case *k*:

3 $$ {p}_{ij}\left(\mathbf{v},\mathbf{w},\boldsymbol{\upvarphi}; \boldsymbol{\uptheta} \right)=\frac{\pi_{ij}\left(\mathbf{v},\mathbf{w},\boldsymbol{\upvarphi} \right)g\left({t}_i-{t}_j\Big|\boldsymbol{\uptheta} \right)}{{\displaystyle {\sum}_{k\ne i}{\pi}_{ik}\left(\mathbf{v},\mathbf{w},\boldsymbol{\upvarphi} \right)g\left({t}_i-{t}_k\Big|\boldsymbol{\uptheta} \right)}} $$

The probability of a potentially infectious contact between *i* and *j*, *π*_*ij*_, is based on the contact information (**v**,**w**) collected during the outbreak and the types **φ** of infection of both cases *i* and *j*. To distinguish different types of infection and to reflect the fact that there is only limited mixing between students in building 1 and building 2, we define the following,

4 $$ \begin{array}{l}{\psi}_{ij}=1\kern0.75em \mathrm{if}\ \mathrm{c}\mathrm{a}\mathrm{se}\kern0.5em i\kern0.5em \mathrm{a}\mathrm{nd}\ \mathrm{c}\mathrm{a}\mathrm{se}\kern0.5em j\kern0.5em \mathrm{r}\mathrm{e}\mathrm{side}\ \mathrm{in}\ \mathrm{the}\ \mathrm{same}\ \mathrm{dormitory}\ \mathrm{a}\mathrm{nd}\kern0.5em \mathrm{a}\mathrm{r}\mathrm{e}\kern0.5em \mathrm{the}\ \mathrm{same}\ \mathrm{type}\ \mathrm{o}\mathrm{f}\ \mathrm{in}\mathrm{f}\mathrm{e}\mathrm{c}\mathrm{tion}\ \mathrm{o}\mathrm{r}\ \mathrm{if}\ \mathrm{c}\mathrm{a}\mathrm{se}\kern0.5em j\kern0.5em \mathrm{is}\kern0.5em \mathrm{a}\ \mathrm{c}\mathrm{o}-\mathrm{infection};\\ {}{\psi}_{ij}=w\kern0.75em \mathrm{if}\ \mathrm{c}\mathrm{a}\mathrm{se}\kern0.5em i\kern0.5em \mathrm{a}\mathrm{nd}\ \mathrm{c}\mathrm{a}\mathrm{se}\kern0.5em j\kern0.5em \mathrm{r}\mathrm{e}\mathrm{side}\ \mathrm{in}\ \mathrm{different}\ \mathrm{dormitories}\ \mathrm{a}\mathrm{nd}\kern0.5em \mathrm{a}\mathrm{r}\mathrm{e}\kern0.5em \mathrm{the}\ \mathrm{same}\ \mathrm{type}\ \mathrm{o}\mathrm{f}\ \mathrm{in}\mathrm{f}\mathrm{e}\mathrm{c}\mathrm{tion}\ \mathrm{o}\mathrm{r}\ \mathrm{if}\ \mathrm{c}\mathrm{a}\mathrm{se}\kern0.5em j\kern0.5em \mathrm{is}\kern0.5em \mathrm{a}\ \mathrm{c}\mathrm{o}-\mathrm{infection};\\ {}{\psi}_{ij}=0\kern0.5em \mathrm{o}\mathrm{the}\mathrm{r}\mathrm{wise}.\end{array} $$

**Fig. 1:**
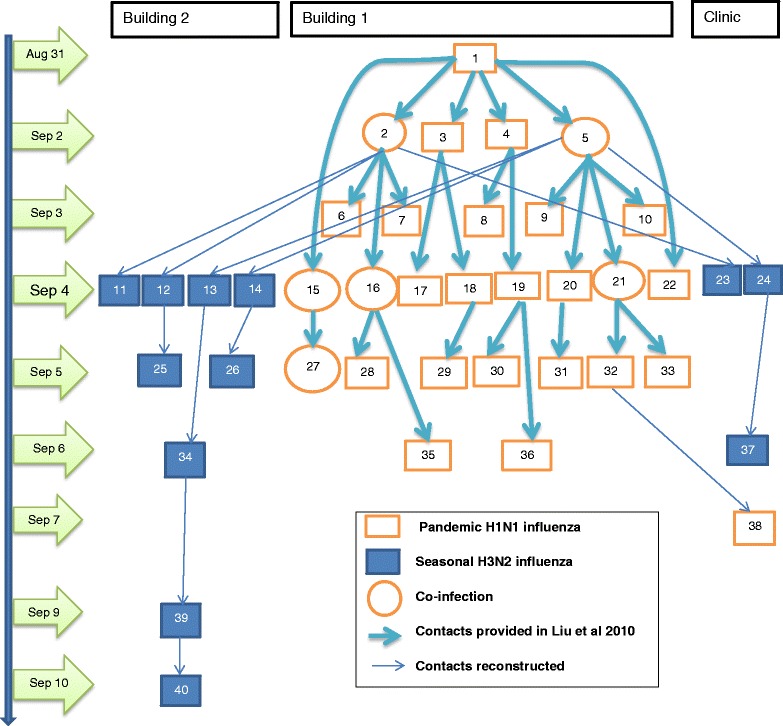
One plausible transmission tree. The tree was constructed under the assumption of limited mixing rate between students in two dormitories in relation to within building mixing rates (*w* = 25 %). In the construction, the other possible sources of seasonal A/H3N2 virus (e.g., the two question marked boxes above building 2 and clinic in Fig. 2 of Liu et al. [12]) have been ignored. The thick arrows represent transmissions from Liu et al. [12] while the thin arrows display one of the most likely transmissions

The variable *w* defines the extent of contact rates between students in two dormitories in relation to contact rates within dormitories: *w* = 0 implies that contacts between dormitories are forbidden and *w* = 1 suggests that contacts between dormitories are the same as that within each dormitory. Student access to the college clinic was not restricted. The original numberings of patients in Fig. 2 of Liu et al. [12] are given in accordance with the order of symptom onset within each building. For the convenience of our analysis, the forty patients have been re-indexed in the order of symptom onset as 1, 2, …, 40 (see Fig. 1). If cases *i* and *j* form a likely transmission pair (i.e., *v*(*i*) = *j*) and there is only one possible infector, *π*_*ij*_(**v**,**w,φ**) = *ψ*_*ij*_ and *p*_*ij*_(**v**,**w,φ;θ**) =1. If the outbreak investigation reveals that case *i* is not the index case and does not contact any of the *i*-1 cases that developed symptoms before case *i*, the probability of a potentially infectious contact is *π*_*ij*_(**v**,**w,φ**) = *ψ*_*ij*_/*η*_*i*-1_ Here *η*_*i*-1_ is the ‘effective’ number of infections that developed symptoms before case *i* and are of the same type of infection as case *i* or co-infection, and is calculated as *η*_*i* − 1_ = ∑_*k* = 1_^*i* − 1^ *ψ*_*ik*_.

Given the contact information (**v**,**w**), the most likely transmission tree can be obtained by finding the values of parameters **θ** = {*a*, *b*} that maximize the total log-likelihood (1). The downhill simplex method [16] was used to locate the maximum-likelihood estimate (MLE), $\widehat{\boldsymbol{\uptheta}}$, of the parameters. We also used the MCMC method to sample the values of parameters **θ** and find their medians, 2.5 percentile and 97.5 percentile.

From the probabilities that case *i* is infected by case *j*: {*p*_1*i*_, …, *p*_*Ni*_}, we can sample a transmission tree in which all cases are connected and case 1 is the index case (Fig. 1) and further estimate transmissibility. The case reproduction number is the average number of secondary cases generated by primary cases, which measures the transmissibility of infection. The overall case-reproduction number on day *t*, *R*_*t*_, can be estimated by summing over all these infectious contacts:

5 $$ {R}_t={\displaystyle \sum_j{\displaystyle \sum_{i=2}{p}_{ij}}}\left(\mathbf{v},\mathbf{w},\boldsymbol{\upvarphi}; \widehat{\boldsymbol{\uptheta}}\right) $$

We can further estimate the infection type-specific reproductive numbers when both case *i* and case *j* are of the same type of infection:

6 $$ \begin{array}{l}{R}_{\mathrm{e}}^1={\displaystyle \sum_j{\displaystyle \sum_{i=2}{p}_{ij}\left(\mathbf{v},\mathbf{w},\boldsymbol{\upvarphi}; \widehat{\boldsymbol{\uptheta}}\right){\delta}_{ij;\mathrm{H}1\mathrm{N}1}}}\\ {}{R}_{\mathrm{e}}^2={\displaystyle \sum_j{\displaystyle \sum_{i=2}{p}_{ij}\left(\mathbf{v},\mathbf{w},\boldsymbol{\upvarphi}, \widehat{\boldsymbol{\uptheta}}\right){\delta}_{ij;\mathrm{H}3\mathrm{N}2}}}\\ {}{R}_{\mathrm{e}}^{\mathrm{C}}={\displaystyle \sum_j{\displaystyle \sum_{i=2}{p}_{ij}\left(\mathbf{v},\mathbf{w},\boldsymbol{\upvarphi}; \widehat{\boldsymbol{\uptheta}}\right){\delta}_{ij;\mathrm{c}\mathrm{o}\hbox{-} \mathrm{infection}}}}\end{array} $$

Here the symbol *δ*_*ij*,TYPE_ is defined as: *δ*_*ij*,TYPE_ =1 if both cases *i* and *j* are of the infection TYPE; *δ*_*ij*,TYPE_ = 0 otherwise. To characterise the interaction between two types of virus within co-infected individuals, we further estimate the reproductive numbers of A/H1N1 and A/H3N2 due to co-infected individuals as:

7 $$ \begin{array}{l}{R}_{\mathrm{e},\mathrm{C}}^1={\displaystyle \sum_j{\displaystyle \sum_{i=2}{p}_{ij}\left(\mathbf{v},\mathbf{w},\boldsymbol{\upvarphi}; \widehat{\boldsymbol{\uptheta}}\right){\varDelta}_{ij;\mathrm{H}1\mathrm{N}1}}}\\ {}{R}_{\mathrm{e},\mathrm{C}}^2={\displaystyle \sum_j{\displaystyle \sum_{i=2}{p}_{ij}\left(\mathbf{v},\mathbf{w},\boldsymbol{\upvarphi}; \widehat{\boldsymbol{\uptheta}}\right){\varDelta}_{ij;\mathrm{H}3\mathrm{N}2}}}\end{array} $$

Here ∆ _*ij*,TYPE_ is defined as: ∆ _*ij*,TYPE_ =1 if infector case *j* is co-infected while the infectee case *i* is singly infected with type TYPE; otherwise ∆ _*ij*,TYPE_ =0. The strain interactions within co-infections are estimated as:

8 $$ \begin{array}{l}{\varphi}_1=\frac{R_{\mathrm{e},\mathrm{C}}^1}{R_{\mathrm{e}}^1}\\ {}{\varphi}_2=\frac{R_{\mathrm{e},\mathrm{C}}^2}{R_{\mathrm{e}}^2}\end{array} $$

Here *ϕ*_1_ measures the effect on infectivity of A/H1N1 within co-infections and *ϕ*_2_ measures the effect on infectivity of A/H3N2 within co-infections.

### Ethics considerations

Data that were used in the analysis of this study were extracted from a previous study [12] and hence did not require Human Resource Ethics committee approval.

## Results and discussion

The data shown in Fig. 2 of Liu et al. [12] indicates that the index case is infected with pandemic A/H1N1 virus. Among the six cases that the index case infects, three patients (i.e., case 2, 5, and 15) are co-infected with both A/H1N1 and A/H3N2. It was assumed that seasonal A/H3N2 virus was endemic [12], however, the infectious contacts with A/H3N2 of these three co-infected cases were unknown. Several possible scenarios are possible: cases 2, 5 and 15 were exposed to pandemic A/H1N1 virus when they were still infectious with A/H3N2; cases 2, 5 and 15 were further exposed to seasonal A/H3N2 virus soon after they had acquired pandemic A/H1N1 virus from the index case; the three cases were infected simultaneously with both A/H1N1 and A/H3N2 viruses from the index case who was actually co-infected before contact with cases 2, 5 and 15 but was incorrectly typed as a single infection with pandemic A/H1N1 virus. For simplicity, we use all the contact information for cases within building 1, especially the pathways from the index case to the three co-infections, ignoring the possible pathways for transmitting background endemic seasonal A/H3N2 virus.

**Fig. 2:**
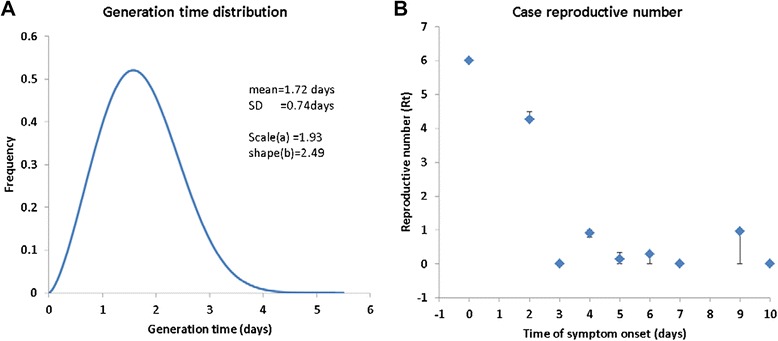
Constructed transmission tree of influenza A virus: **a** The relative frequency of the generation intervals; **b** The average case reproduction number *R*_*t*_ as it varies with time. Bars represent 95 % nonparametric bootstrap percentile confidence intervals generated from one million possible transmission trees sampled from the contact probabilities listed in Table 1

The maximum likelihood estimates of the infective contact probabilities *p*_*ij*_(**v**,**w,φ;θ**) are listed in Table 1 from which one sample transmission tree is shown in Fig. 1. The MLEs of the generation interval distribution *g*(∆*t*|**θ**) and the time course of the mean case reproductive number *R*_*t*_ are shown in Fig. 2. The generation interval has a mean of 1.72 days and a standard deviation (SD) of 0.74 days. The mean case reproductive number over the whole outbreak is 1.57 with SD = 0.022. Before the isolation of cases and the initiation of prophylaxis among the campus population occurred (5 September 2009), the reproductive number is estimated to be 2.80 with SD = 0.016; after this it declines to 0.341 with SD = 0.047. This clearly shows the effectiveness of isolation and prophylaxis.

**Table 1:** The most likely contact probabilities under the limited mixing between dormitories (*w* = 25 %)

Infectee *i*	Infector *j*	*p*_*ij*_	Infectee *i*	Infector *j*	*p*_*ij*_
1 (1)	--		35 (1)	16 (0)	1.0
2 (0)	1 (1)*	1.0	36 (1)	19 (1)	1.0
3 (1)	1 (1)	1.0	37 (2)	2 (0)	0.001602
4 (1)	1 (1)	1.0	37 (2)	5 (0)	0.001602
5 (0)	1 (1)*	1.0	37 (2)	11 (2)	0.08588
6 (1)	2 (0)	1.0	37 (2)	12 (2)	0.08588
7 (1)	2 (0)	1.0	37 (2)	13 (2)	0.08588
8 (1)	4 (1)	1.0	37 (2)	14 (2)	0.08588
9 (1)	5 (0)	1.0	37 (2)	15 (0)	0.08588
10 (1)	5 (0)	1.0	37 (2)	16 (0)	0.08588
11 (2)	2 (0)	0.5	37 (2)	21 (0)	0.08588
11 (2)	5 (0)	0.5	37 (2)	23 (2)	0.08588
12 (2)	2 (0)	0.5	37 (2)	24 (2)	0.08588
12 (2)	5 (0)	0.5	37 (2)	25 (2)	0.074625
13 (2)	2 (0)	0.5	37 (2)	26 (2)	0.074625
13 (2)	5 (0)	0.5	37 (2)	27 (0)	0.074625
14 (2)	2 (0)	0.5	38 (1)	1 (1)	3.67E-11
14 (2)	5 (0)	0.5	38 (1)	2 (0)	2.53E-05
15 (0)	1 (1)*	1.0	38 (1)	3 (1)	2.53E-05
16 (0)	2 (0)	1.0	38 (1)	4 (1)	2.53E-05
17 (1)	3 (1)	1.0	38 (1)	5 (0)	2.53E-05
18 (1)	3 (1)	1.0	38 (1)	6 (1)	0.001691
19 (1)	4 (1)	1.0	38 (1)	7 (1)	0.001691
20 (1)	5 (0)	1.0	38 (1)	8 (1)	0.001691
21 (0)	5 (0)	1.0	38 (1)	9 (1)	0.001691
22 (1)	1 (1)	1.0	38 (1)	10 (1)	0.001691
23 (2)	2 (0)	0.5	38 (1)	15 (0)	0.024945
23 (2)	5 (0)	0.5	38 (1)	16 (0)	0.024945
24 (2)	2 (0)	0.5	38 (1)	17 (1)	0.024945
24 (2)	5 (0)	0.5	38 (1)	18 (1)	0.024945
25 (2)	2 (0)	0.011463	38 (1)	19 (1)	0.024945
25 (2)	5 (0)	0.011463	38 (1)	20 (1)	0.024945
25 (2)	11 (2)	0.144752	38 (1)	21 (0)	0.024945
25 (2)	12 (2)	0.144752	38 (1)	22 (1)	0.024945
25 (2)	13 (2)	0.144752	38 (1)	27 (0)	0.090627
25 (2)	14 (2)	0.144752	38 (1)	28 (1)	0.090627
25 (2)	15 (0)	0.036188	38 (1)	29 (1)	0.090627
25 (2)	16 (0)	0.036188	38 (1)	30 (1)	0.090627
25 (2)	21 (0)	0.036188	38 (1)	31 (1)	0.090627
25 (2)	23 (2)	0.144752	38 (1)	32 (1)	0.090627
25 (2)	24 (2)	0.144752	38 (1)	33 (1)	0.090627
26 (2)	2 (0)	0.011463	38 (1)	35 (1)	0.07875
26 (2)	5 (0)	0.011463	38 (1)	36 (1)	0.07875
26 (2)	11 (2)	0.144752	39 (2)	2 (0)	1.70E-10
26 (2)	12 (2)	0.144752	39 (2)	5 (0)	1.70E-10
26 (2)	13 (2)	0.144752	39 (2)	11 (2)	0.00047
26 (2)	14 (2)	0.144752	39 (2)	12 (2)	0.00047
26 (2)	15 (0)	0.036188	39 (2)	13 (2)	0.00047
26 (2)	16 (0)	0.036188	39 (2)	14 (2)	0.00047
26 (2)	21 (0)	0.036188	39 (2)	15 (0)	0.000118
26 (2)	23 (2)	0.144752	39 (2)	16 (0)	0.000118
26 (2)	24 (2)	0.144752	39 (2)	21 (0)	0.000118
27 (0)	15 (0)	1.0	39 (2)	23 (2)	0.00047
28 (1)	15 (0)	0.5	39 (2)	24 (2)	0.00047
28 (1)	16 (0)	0.5	39 (2)	25 (2)	0.031389
29 (1)	16 (0)	0.5	39 (2)	26 (2)	0.031389
29 (1)	16 (0)	0.5	39 (2)	27 (2)	0.007847
30 (1)	19 (1)	1.0	39 (2)	34 (2)	0.4631
31 (1)	19 (1)	0.5	39 (2)	37 (2)	0.4631
31 (1)	20 (1)	0.5	40 (2)	2 (0)	8.39E-15
32 (1)	21 (0)	1.0	40 (2)	5 (0)	8.39E-15
33 (1)	21 (0)	1.0	40 (2)	11 (2)	8.98E-07
34 (2)	2 (0)	0.000535	40 (2)	12 (2)	8.98E-07
34 (2)	5 (0)	0.000535	40 (2)	13 (2)	8.98E-07
34 (2)	11 (2)	0.114752	40 (2)	14 (2)	8.98E-07
34 (2)	12 (2)	0.114752	40 (2)	15 (0)	2.24E-07
34 (2)	13 (2)	0.114752	40 (2)	16 (0)	2.24E-07
34 (2)	14 (2)	0.114752	40 (2)	21 (0)	2.24E-07
34 (2)	15 (0)	0.028688	40 (2)	23 (2)	8.98E-07
34 (2)	16 (0)	0.028688	40 (2)	24 (2)	8.98E-07
34 (2)	21 (0)	0.028688	40 (2)	25 (2)	0.000308
34 (2)	23 (2)	0.114752	40 (2)	26 (2)	0.000308
34 (2)	24 (2)	0.114752	40 (2)	27 (0)	7.71E-05
34 (2)	25 (2)	0.099713	40 (2)	34 (2)	0.020572
34 (2)	26 (2)	0.099713	40 (2)	37 (2)	0.020572
34 (2)	27 (0)	0.024928	40 (2)	39 (2)	0.958157

The numbers in the brackets are the type of infection: 0, 1, 2 representing co-infection, infection with pandemic A/H1N1 virus alone and infection with seasonal A/H3N2 virus alone, respectively. The three effective contacts from the index pandemic A/H1N1 to co-infection that are marked with asterisk are as given by Liu et al. [12]. The maximum likelihood estimates of Weibull distribution parameters for the generation time are scale =1.93 and shape = 2.49

To measure the interactions between two influenza A viruses, we first estimated the values of infection type-specific reproductive numbers before the initiation of isolation and prophylaxis occurred. Because most transmissions that involve A/H1N1 and co-infections are known, the estimates of the infection type-specific reproductive numbers are stable at *R*_*e*_^1^ = 1.44 and *R*_*e*_^*C*^ = 0.67, and the reproductive number of A/H1N1 due to co-infection is *R*_*e*,*C*_^1^ = 2.01. In contrast, transmissions that involve A/H3N2 were missing and so estimates of their reproductive numbers show some uncertainty: *R*_*e*_^2^ has a mean 0.48 and 95 % confidence interval of [0.45, 0.51], and the reproductive number of A/H3N2 due to co-infection is *R*_*e*,*C*_^2^ = 1.60 [1.58, 1.62]. Although the estimate of *R*_*e*_^1^ for A/H1N1 is in agreement with the usual estimates [15, 17], that of *R*_*e*_^2^ for A/H3N2 is much smaller than other estimates which range from 1.19 to 1.37 [17]. The likely reason for this difference lies in the fact that pandemic A/H1N1 is a novel virus while A/H3N2 is an endemic seasonal virus in the study region [12] so some pre-existing immunity against A/H3N2 due to the previous infections and/or vaccination reduces its susceptibility and hence its spread. In the presence of co-infection, infectivity of A/H1N1 increases moderately with *ϕ*_1_ = 1.4. However, the increased infectivity of A/H3N2 in the presence of co-infection is large: *ϕ*_2_ = 3.28 [95 % CI: 3.18—3.38] and so co-infection is estimated to contribute greatly to the outbreak. This is not inconsistent with Liu et al. [12]’s observation that “no patients had detectable hemagglutination inhibiting antibodies against pandemic H1N1 virus in their acute-phase samples”. Although the presence of co-infection can increase the infectivity of each component influenza virus, co-infection itself cannot spread successfully because the co-transmissibility measured by *R*_*e*_^*C*^ is estimated to be 0.67, which is less than one.

The above results are obtained under the assumption of *w* = 25 %, which is used to reflect the observation “although there were activities outside the dormitory, the members of the 2 buildings did not interact with each other to any significant extent” (p1361, second column, Liu et al. [12]). If different values of *w* are chosen, the estimation of transmissibility involved with A/H3N2 virus changes although the results of the overall transmissibility and those involved with A/H1N1 remain nearly the same. With a low value of *w* (i.e. the mixing between two dormitories become more restrictive), the reproductive number of A/H3N2 due to co-infection (*R*_*e*,*C*_^2^) decreases while *R*_*e*_^2^ increases; this consequently reduces the value of *ϕ*_2_. For example, the estimate of *ϕ*_2_ becomes 3.00 [2.92, 3.13] when *w* =0.1. This is the outcome because without the index case of A/H3N2 infection, the only possible infectors of A/H3N2 cases 11, 12, 13, 14 in building 2 are co-infection cases 2 and 5, which is independent of the limited mixing rate between two dormitories (*w*). In the situation without limitation in mixing rates between two dormitories (i.e., *w* = 1.0), the estimate of *ϕ*_2_ is 4.57 [4.42, 4.70]. This result implies that in the absence of any unidentified index cases of A/H3N2 infection, co-infection can enhance the transmissibility of each component virus.

However, all the cases reported in building 2 were infections with A/H3N2 virus alone. In view of the restrictive mixing between two dormitories, it is likely that there is an unknown index case of A/H3N2 infection within building 2, although it was not reported. An analysis listed in the Additional file 1 shows that such a hidden index case could change the above conclusion: the reproductive number of A/H3N2 due to co-infection (*R*_*e*,*C*_^2^) might not exceed the reproductive number by its own (*R*_*e*_^2^) and therefore co-infection could not enhance the infectivity of A/H3N2. Jombart et al. [18] have developed a Bayesian method to reconstruct disease outbreaks by combining epidemiological and genomic data. This may allow for the tests of whether co-infection cases in building 1 are the infectors of A/H3N2 cases in building 2 or whether there is a hidden index case of A/H3N2 infection in building 2. Unfortunately, the representative sequences deposited in GenBank by Liu et al. [12] were not complete genome sequences and were not marked with the relevant symptom onset information. Hence they cannot help to distinguish and/or find the potential sources of A/H3N2 infection in the transmission tree. Conditional on the available information, the evidence about how co-infection alters the infectivity of A/H3N2 virus is lacking. Nevertheless, the conclusion about enhanced infectivity of A/H1N1 within co-infection is not affected.

The original method of reconstructing the transmission tree by Hens et al. [14] relies on three assumptions: all cases are observed; all of them except the index case are infected by another observed case; and the generation interval distribution remains unchanged. To apply to such outbreaks as studied here involving two viruses, some assumptions have been strengthened. There must be two index cases that were singly infected with different viruses or one index case that was co-infected with the two viruses. There are more than one transmission processes. In our study example, there are five transmission processes: A/H1N1 to A/H1N1; A/H3N2 to A/H3N2; co-infection to A/H1N1; co-infection to A/H3N2 and co-infection to co-infection. Unfortunately the available data does not provide direct information for all the transmission processes, and it is not directly possible to assess the heterogeneity in generation intervals among transmission processes. In view of the similar estimates for generation intervals of different transmission processes [15], the generation interval distribution has been assumed to be unchanged over different types of infection as well as over the course of the outbreak.

Another aspect is how co-infection is generated. It could be due to an infection with one virus becoming a co-infection, or it could be a consequence of a co-transmission. And it is possible that going from A/H1N1 to co-infection is easier than going from A/H3N2 to co-infection, or vice versa. Though this is an interesting issue [19] the limited information that we can collect from Liu et al. [12] cannot allow us to detect the order in which the two viruses are acquired by an co-infected individual and therefore no way to investigate the effect of the order in which the two viruses are acquired.

## Conclusion

Reconstructing transmission trees provides useful information about generation interval and transmission rate of infectious diseases, which are important for designing containment strategies. In this study, the method of reconstructing the plausible transmission tree from the incomplete data of an outbreak caused by one virus (Hens et al. [14]) has been extended to the outbreak caused by two influenza A viruses. Our estimates of epidemiological characteristics such as the generation interval and the transmission rate of influenza A virus are well within the ranges estimated by others [15, 17]. Our estimation shows that although co-infection with A/H1N1 and A/H3N2 viruses cannot be sustained by co-transmission, it enhances the single transmission of both viruses. However, the concluded enhancement of A/H3N2 virus infectivity within co-infection should be taken with caution owing to the unknown infection source of seasonal H3N2 virus.

Cross-immunity, which characterises the interaction between different viruses when one virus re-infects individuals recovered from previous infection with another virus, has been well recognized and measured. Due to their relative rareness, co-infection and interactions between viruses within co-infections have not yet attracted the attention they deserve. To our knowledge this is the first analysis that estimates the interactions between influenza A viruses within co-infection. Theoretical modelling illustrates their potential role in generating the recurrent epidemics and alternation of the dominant virus in seasonal influenza [20, 21]. Surely this urges more empirical studies to investigate this important issue of influenza and other infectious diseases caused by multiple strains.

## Additional file

Additional file 1:
**Effect of an (unidentified) index case of A/H3N2 infection in building 2.** (DOCX 186 kb)

## Abbreviations

ILI: influenza like illness
MLE: maximum likely estimate
SD: standard deviation
