# Supplementary material for: Construction of the influenza A virus transmission tree in a college-based population: co-transmission and interactions between influenza A viruses
Source: BMC Infect Dis. 2016 Jan 29;16:38. doi: 10.1186/s12879-016-1373-x (PMC4731987; doi:10.1186/s12879-016-1373-x)
Supplement: Additional file 1: — Effect of an (unidentified) index case of A/H3N2 infection in building 2. (DOCX 186 kb) [file 12879_2016_1373_MOESM1_ESM.docx]

**Additional file 1** **Effect of an (unidentified) index case of A/H3N2 infection in building 2**

Although seasonal A/H3N2 virus was prevailing in the college when the pandemic A/H1N1 was introduced into the campus, the exact source of A/H3N2 has not been determined (Liu et al [12]). In this appendix, we assume that there was an unidentified index case of A/H3N2 infection which occurred in building 2 (naming it case ***Q***) and its symptom can onset on any day from 31 August to 3 September (i.e. from the day of symptom onset of case 1 in building 1 to the day just before the day of symptom onset of cases 11, 12, 13, 14 in building 2). We consider two levels of mixing rate between students in two dormitories in relation to within building mixing rate: *w*= 1 and 0.25.

With inclusion of case ***Q*** but no limitation in mixing rates between two buildings (*w*=1, Table A1), the mixed cases 15, 16, 21 and A/H3N2 cases 11, 12, 13, 14, 23, 24 are still equally likely the infectors of A/H3N2 cases 25, 26, 34, 37, 39 and 40. For H3N2 cases 11,12,13,14, 23, and 24, the mixed cases 2 and 5 more likely act as their infectors than case ***Q*** when symptom of case ***Q*** onsets earlier than 2 September; they are equally likely infectors if symptom of case ***Q*** onsets 2 September; case ***Q*** is a more likely infector if its symptom onsets on 3 September.

With inclusion of case ***Q*** and limitation in mixing rates between two buildings (*w*=0.25, Table A2), A/H3N2 cases 11, 12, 13, 14, 23, 24 act more likely as the infectors of H3N2 cases 25, 26, 34, 39 and 40 in building 2 than the mixed cases 15, 16, 21 in building 1. The mixed cases 2 and 5 in building 1 act less likely as the infectors of A/H3N2 cases 11, 12, 13, and 14 than case ***Q*** if symptom of case ***Q*** onsets later than 31 August.

A most likely transmission tree when *w*=0.25 is shown in Figure A1. Inclusion of case ***Q*** does not affect the estimation of $R_{e}^{1}$, $R_{e,C}^{1}$(and thus *φ*_1_) that relate to A/H1N1, and slightly changes the generation interval distribution. For example, when varying the symptom onset date of case ***Q*** from 31 August to 3 September, the mean generation interval ranges from 1.62 to 1.72 days and SD from 0.71 to 0.74 days. However, inclusion of case ***Q*** substantially changes the estimates of A/H3N2-related and overall reproductive numbers (Table A1 and Table A2). Figure A2 shows how the day of symptom onset of case ***Q*** alters the time course of the overall case reproductive number. With the early occurrence of case ***Q*** (before 1 September), $R_{e}^{2}$still remains low and$R_{e,C}^{2}$high because the contribution of case ***Q*** is small, which leads to the estimate of *φ*_2_ being greater than one. When case ***Q*** occurs later (after 2 September), its contribution increases to an extent so that $R_{e}^{2}$exceeds $R_{e,C}^{2}$ which gives rise to *φ*_2_ <1 (Table A1). The limited mixing between two dormitories further reduces the estimated value of *φ*_2_ (Table A2). However, the true circumstances might not allow this to take place. In view of what Liu et al said (page1361 [12]) “most patients with ILI sought medical care in the college clinic”, anyone with ILI on or after 2^nd^ September, the day when cases 2, 3, 4, 5 have symptom onset, will very likely visit the college clinic for medical help. This implies that symptom onset for the unidentified A/H3N2 case ***Q*** is very likely before 2^nd^ September unless its symptoms were very mild. This argument lends support for the enhanced transmissibility of A/H3N2 in co-infection.

Figure legends

**Figure A1** One plausible transmission tree. The tree was constructed under the assumption of a limited mixing rate between students in two dormitories in relation to within building mixing rates (*w*=25%) and with inclusion of an unidentified index case of A/H3N2 in building 2, whose symptom onset on 1 September 2009 (marked as ***Q***). The thick arrows represent transmissions from Liu et al. [12] while the thin arrows display one of the most likely transmissions. Comparison with Figure 1 in the main text shows that case ***Q*** substantially decreases the contribution of co-infections (i.e., cases 2, 5, 15, 16, and 22) in generating secondary cases of A/H3N2 infection alone in building 2. Meanwhile, it increases the reproductive number of A/H3N2 by its own. Hence this reduces the possibility that co-infection increases the transmissibility of A/H3N2 virus.

**Q**

Clinic

Building 2

Building 1

Sep 7

Sep 10

Sep 9

Sep 3

Sep 6

Sep 5

Aug 31

Sep 2

Sep 4

11

12

13

14

25

40

39

34

26

38

**Pandemic H1N1 influenza**

**Seasonal H3N2 influenza**

**Co-infection**

**Contacts provided in Liu et al 2010**

**Contacts reconstructed**

28

29

19

20

17

18

22

6

24

23

37

30

35

7

3

8

4

1

9

10

31

33

32

36

**Figure A2** The time course of the overall case reproductive number *R*_t_. The four different symptom onset days of case ***Q*** are shown. The bars represent 95% nonparametric bootstrap percentile confidence.


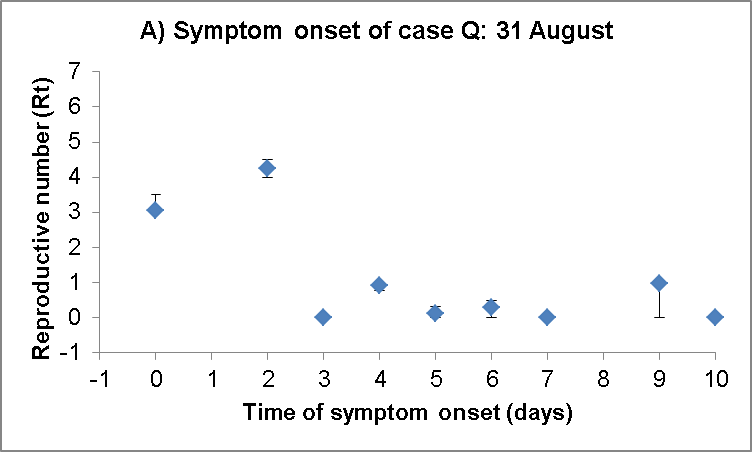

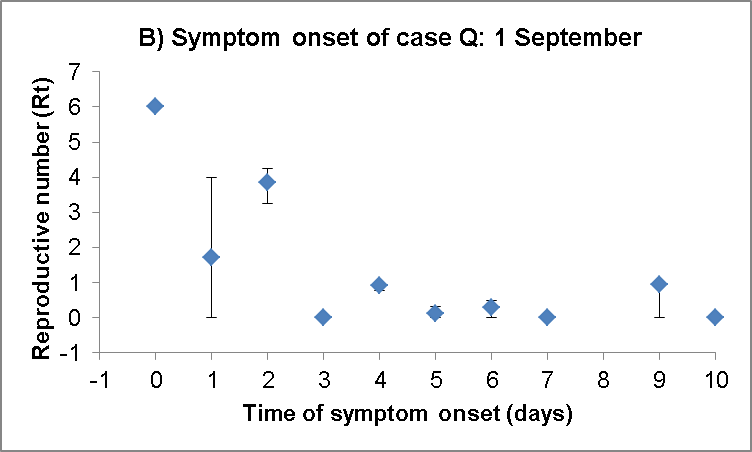

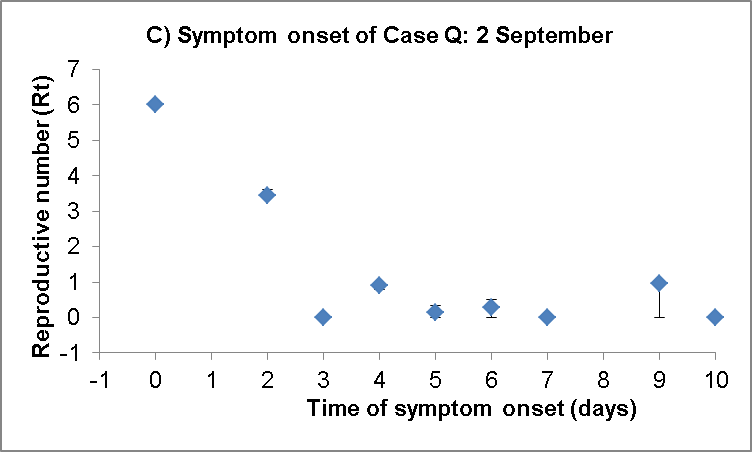

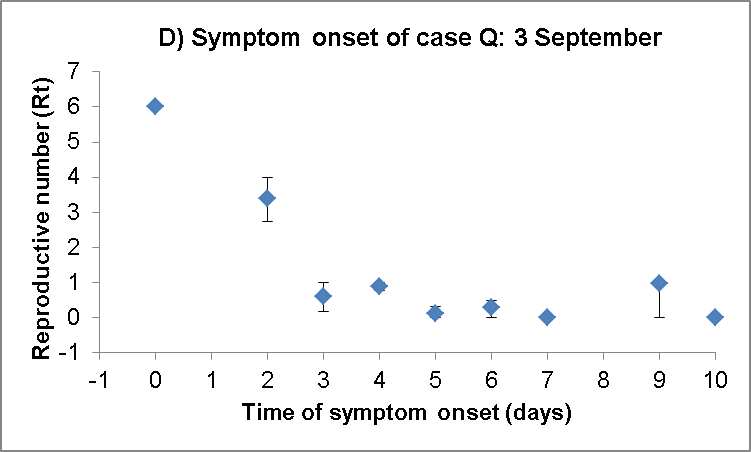


**Table A1** Impact of case ***Q*** without restriction on mixing rate between building (*w*=1).

| Onset of Case Q | R_e_ | | | $R_{e}^{2}$ | $R_{e,C}^{2}$ | *φ*_2_ |
| --- | --- | --- | --- | --- | --- | --- |
|  | Overall | Before control | After control | mean (95%CI) | mean (95%CI) | mean (95%CI) |
| 31 Aug | 1.20(.022) | 2.05(.025) | .343(.044) | .21[.18,.32] | 1.70[1.65,1.73] | 7.70[5.28,8.78] |
| 1 Sep | 1.26(.070) | 2.35(.121) | .341(.046) | .56[.37,.76] | 1.53[1.45,1.59] | 2.80[1.86,4.12] |
| 2 Sep | 1.46(.022) | 2.59(.019) | .341(.046) | 1.21[1.18,1.23] | 1.21[1.18,1.23] | 1.0[0.98,1.0] |
| 3 Sep | 1.55(.025) | 2.76(.033) | .344(.045) | 1.26[1.00,1.57] | 1.20[1.00,1.37] | .93[.62,1.34] |

**Table A2** Impact of case ***Q*** with limited mixing between buildings in relation to within buildings (*w*=0.25).

| Onset of Case Q | R_e_ | | | $R_{e}^{2}$ | $R_{e,C}^{2}$ | *φ*_2_ |
| --- | --- | --- | --- | --- | --- | --- |
|  | Overall | Before control | After control | mean (95%CI) | mean (95%CI) | mean (95%CI) |
| 31 Aug | 1.20(.020) | 2.05(.025) | .346(.039) | .33[.28,.54] | 1.55[1.43,1.59] | 4.88[2.58,6.28] |
| 1 Sep | 1.53(.070) | 2.49(.161) | .341(.048) | 1.1[.73,1.47] | 1.16[.99,1.33] | 1.08[.66,1.77] |
| 2 Sep | 1.46(.021) | 2.58(.018) | .342(.045) | 1.94[1.91,1.98] | .76[.74,.78] | .38[.38,.38] |
| 3 Sep | 1.54(.024) | 2.73(.031) | .347(.044) | 2.06[1.76,2.31] | .73[.54,.91] | .35[.22,.50] |
